# *Stachys pilifera* Benth. Ameliorates Bleomycin-Induced Pulmonary Fibrosis in Rats through the Antioxidant Pathways

**DOI:** 10.1155/2022/6208102

**Published:** 2022-09-30

**Authors:** Nazanin Danaei, Esmaeel Panahi kokhdan, Hossein Sadeghi, Heibatollah Sadeghi, Sajad Hassanzadeh, Davoud Rostamzadeh, Nahid Azarmehr, Salar Hafez Ghoran

**Affiliations:** ^1^Medicinal Plants Research Center, Yasuj University of Medical Sciences, Yasuj, Iran; ^2^Department of Chemistry, Faculty of Science, Golestan University, Gorgan, Iran

## Abstract

**Methods:**

In this experimental study, 35 male Wistar rats (120–180 g) were divided into five groups (*n* = 7) as follows: intratracheal instillation of bleomycin (BLM, 7.5 IU/kg) was administered to group II. The third and fourth groups received BLM plus *Stachys pilifera* hydroalcoholic extract (SPHE) (300 mg/kg/day, gavage). Vitamin *E* (500 mg/kg/day, gavage) was given to group V in addition to BLM. After 14 days, the animals were euthanized to assess biochemical parameters and lung histopathology. Malondialdehyde (MDA), nitric oxide (NO), total thiol (TSH), and glutathione (GSH) levels were measured. In addition, hydroxyproline (HYP) levels along with histological changes in lung tissue were also assessed.

**Results:**

MDA, NO, and HYP elevations induced by BLM toxicity were significantly inhibited by SPHE (300 and 600 mg/kg), and Vit E. SPHE also significantly increased GSH and TSH levels in comparison to the BLM group.HPLC analyses showed the presence of thymol (55.47 ng/mL) and carvacrol (109.91 ng/mL) in SPHE as potential bioactive phenolic compounds.

**Conclusion:**

The results suggest that SPHE alleviates the development of BLM-induced pulmonary fibrosis by inhibiting the proliferation of fibroblasts mediated by antioxidant pathways. Other mechanisms underlying this Effect of SPHE need to be clarified through further research.

## 1. Introduction

Idiopathic pulmonary fibrosis (IPF) is a chronic and progressive lung disease characterized by proliferation and differentiation of fibroblasts and accumulation of extracellular matrix, primarily collagen, leading to irreversible damage to lung architecture [1, 2]. In some acute lung injury survivors, excessive alveolar injury stimulated the proliferation and repair system. As a result, many pulmonary interstitial cells and fibroblasts filled the damaged alveolar interstitium with neovascularization, causing excessive alveolar repair and pulmonary fibrosis [3]. Due to the limited possibility of initial diagnosis and intervention, the average life of patients with IPF is 3 to 5 years after diagnosis. The incidence of IPF is estimated to range from 6.8 to 16.3 per 100,000 [4]. Although the cause of IPF is unknown, environmental pollution, viral infections, genetic factors, inflammation, and oxidative stress are involved [5]. In addition, some drugs, such as bleomycin (BLM), methotrexate, and nitrofurantoin, induce pulmonary fibrosis [6]. Lungs are more exposed to exogenous oxidants than other organs due to their unique position and function. Antioxidants in epithelial lining fluid counteract these oxidants. However, this defense system can be disrupted by the overproduction of reactive oxygen species (ROS) and reactive nitrogen species (RNS) in disease states. In order to reduce the toxicity caused by ROS, enzymatic antioxidants such as GPX, SOD, and catalase and molecular antioxidant systems such as Vit C and Vit *E* are activated [7]. Other antioxidants include thiol-containing compounds such as glutathione (GSH) and thioredoxin [8]. Numerous studies have proven the role of oxidative stress in the pathogenesis of IPF [5, 9, 10]. Increased levels of oxidative stress markers have been shown in patients with pulmonary fibrosis [5].

Bleomycin (BLM) is a chemotherapeutic antibiotic used to treat a variety of malignancies. Since one of the most crucial side effects of BLM during the chemotherapy process is pulmonary fibrosis, it is a classic combination for modeling pulmonary fibrosis in laboratory animals [6, 11]. Intratracheal BLM injection causes interstitial and alveolar inflammation, macrophage activation, and increased inflammatory factors. It also stimulates ROS production, including superoxide and hydroxyl radicals [12]. There is currently no effective treatment for most types of IPF [1]. For example, using methylprednisolone effectively improves BLM-induced IPF, but it has disadvantages such as inhibiting the immune system. Therefore, new therapeutic compounds with minimal side effects for preventing and treating PF have been considered [10].

*Stachys pilifera* is a perennial and fragrant plant belonging to the Lamiaceae family [13]. In traditional medicine, some *Stachys* species have been utilized as antinephritis, antidiarrhea, and wound disinfectants [14, 15]. Moreover, the antibacterial, antioxidant, and cytotoxic effects of some *Stachys* species have been confirmed [16]. Different species of this genus contain phytochemicals such as flavonoids, iridoids, diterpenoids, and phenolic acids [17]. Farjam et al. used the DPPH test to prove the antioxidant properties of *S. pilifera* [18]. In our previous studies, the effects of hepatic and renal protection of the plant were shown by reducing oxidative stress [19, 20]. Hence, the main objective of this study was to evaluate the protective effect of *S. pilifera* hydroalcoholic extract (SPHE) on bleomycin-induced pulmonary fibrosis in rats.

## 2. Materials and Methods

### 2.1. Chemicals and Reagents

Bleomycin (BLM), 2,4,6-tri(2-pyridyl)-1,3,5-triazine (TPTZ), sulfanilamide, n-(1-naphthyl) ethylenediamine dihydrochloride, thiobarbituric acid (TBA), trichloroacetic acid (TCA), ethylenediaminetetraacetic acid (EDTA) and 5,5′-dithiobis (2-nitrobenzoic acid) (DTNB) were purchased from Merk- Germany. Standards of thymol and carvacrol were obtained from Sigma Aldrich Co. (HPLC grade; ≥ 98% purity; St. Louis, Mo, USA). Acetonitrile (MeCN; HPLC grade) was purchased from Merck, Germany.

### 2.2. Plant Materials

The aerial parts of *Stachys pilifera* were collected from the Kakan region in Kohgiluyeh and Boyer-Ahmad Province in spring 2018. Dr. Azizollah Jafari confirmed the collected samples from the Department of Botany, Center for Research in Natural Resource and Animal Husbandry, Yasuj University, Yasuj, Iran, where a voucher specimen (herbarium No. 1897) was deposited. The plant materials were transferred to the laboratory and dried in a dark place.

### 2.3. Extract Preparation

The air-dried powdered plant (100 g) was macerated in 70% methanol (500 mL) and kept at room temperature for 48 hours (three times 500 mL each). Having filtrated the extract by Whatman No. 1, the solvent was evaporated by rotary apparatus to yield a dark green gummy residue. The extract was stored in the refrigerator at 4°C for further experiments. Briefly, a portion of the extract was dissolved in methanol/water using sonication and stored in a cold place [21, 22]. The filtrated extract was defatted using *n*-hexane through the liquid-liquid extraction (LLE) method. The remained fraction defatted was stored in the refrigerator for technical HPLC analysis.

### 2.4. Quantitative Reverse-phase HPLC Analysis of a Defatted *Stachys pilifera* Portion

In order to calculate the amount of thymol and carvacrol as simple phenolics in SPHE, a high-performance liquid chromatography (HPLC; Model KNAUER®) apparatus was applied equipped with a quaternary K-1001 controller pumps and a K-2550 UV absorbance detector. A binary mobile phase consisting of 45% water and 55% MeCN was gone through a stainless steel analytical column (Zorbax SB-C_18_; 250 mm × 3.9 mm i.d., 5 *μ*m). Moreover, the flow rate and the UV-Vis light detector were set at 1.1 mL/min and 220 nm, respectively. In order to have a calibration curve, about 2 mg of standard thymol and carvacrol (each 1 mg) was dissolved in methanol (HPLC grade) to reach the final concentration of 2000 *μ*g/mL. The calibration curves for standards were prepared using concentrations of 0.5 to 2000 *μ*g/mL [23].

### 2.5. Laboratory Animals

Fifty male Wistar rats 120–180 g were purchased from Razi Institute in Shiraz. Rats were kept in an animal laboratory at a controlled temperature (22 ± 2°C) with a 12-hour light/dark-light cycle and access to adequate food and water. All ethical issues approved by the Committee for Supervision of Laboratory Animals were followed in this work. The code of IR.YUMS.REC.1396.124 was enclosed by the ethics committee of Yasuj University of Medical Sciences.

### 2.6. BLM-Induced Lung Fibrosis

Animals were sedated with intraperitoneal injections of ketamine (50 mg/kg) and xylazine (5 mg/kg). A single intratracheal instillation of BLM (7.5 mg/kg, Nippon Kayaku, Japan) was administered to anesthetized rats by placing them on a biased panel and suspending them by their upper canine teeth, followed by a single intratracheal instillation of Next (7.5 mg/kg, Nippon Kayaku, Japan). The placebo group received the same volume of intratracheal normal saline as the BLM group. The animals were euthanized two weeks following PLF induction [24].

### 2.7. Study Design

Out of fifty male Wister rats, 35 were randomly divided into the following 5 groups (*n* = 7):

  Group I received a single intratracheal instillation of normal saline (control group)
  Group II I received a single intratracheal instillation of BLM solution (7.5 IU/kg in 0.9% NaCl) (BLM-treated group)
  Group III was given a single intratracheal instillation of BLM solution (7.5 IU/kg in 0.9% NaCl) and a daily SPHE (300 mg/kg, gavage) for two weeks (BLM/PPX 0.5)
  Group IV received a single intratracheal instillation of BLM solution (7.5 IU/kg in 0.9% NaCl) and a daily SPHE (600 mg/kg, gavage) for two weeks (BLM/PPX 1)
  Group V was given a single intratracheal instillation of BLM solution (7.5 IU/kg in 0.9% NaCl) and a daily Vit *E* (500 mg/kg, gavage) for two weeks (BLM/Vit E)

The number of animals and the dose of drugs were selected according to previous studies [20, 24, 25].

On the 14 days, the rats were anesthetized with diethyl ether, and blood samples were taken from their cardiac. After that, serum was isolated to assess the indicators of oxidative stress. After sacrificing rats, lung tissue was removed, and a portion was kept in 10% formalin for histological evaluation. After homogenizing and centrifuging were used to quantity oxidative stress and hydroxyproline markers.

### 2.8. Evaluation of Oxidative Stress Markers

#### 2.8.1. Determination of MDA

Serum and tissue levels of MDA were determined based on reaction with TBA. In this method, 1 mL of MDA reagent (mixture of TCA and TBA in 0.25% HCl) was mixed with 500 *μ*L of sample in a microtube. The microtubes were placed in Ben Marie for 95 minutes at 95°C and then in ice for 5 minutes. After centrifugation, the adsorption of the supernatant was read at 535 nm [22, 26].

#### 2.8.2. Determination of Nitric Oxide

Since nitric oxide (NO) is an unstable compound rapidly converted to nitrate and nitrite, its direct measurement is a bit difficult, so the amount of nitrite as a stable NO metabolite was measured by the grease method. In summary, 10 *μ*L of serum or tissue homogenate with 100 *μ*L grease reagent (1% sulfanilamide, 0.1% naphthyl ethylene amide, and hydrochloride in phosphoric acid (5.2%)) was transferred to a 96-well plate and placed in the dark for 15 minutes at room temperature. The absorbance of the samples was then read at 541 nm [27].

#### 2.8.3. Determination of Glutathione Concentration

The Tietze method was used to determine glutathione. In this method, an enzyme cycle in which glutathione reductase (GR) plays a significant role is used to quantify the glutathione present [28].

#### 2.8.4. Determination of Total Thiol

The amount of total thiol (TSH) was determined based on the reaction of DTNB with thiol groups and the formation of TNB (2-nitro-5-thiobenzoic acid) and disulfide compounds. Briefly, 10 *μ*L of DTNB, 150 *μ*L of tris-EDTA, and 790 *μ*L of methanol were added to microtubes containing 25 *μ*L of the sample. After incubation at room temperature, the samples were centrifuged, then the adsorption of the supernatant was read at 412 nm, and the amount of TSH was calculated using a molar absorption coefficient of 13,600 M^−1^·cm^−l^ [29].

#### 2.8.5. Determination of Ferric Reducing Ability of Plasma

Benzie and strain method was used to measure ferric reducing antioxidant power (FRAP) activity. In this method, the ability of plasma to reduce ferric ions is measured. Following the reduction of ferric (Fe^3+^) ions and their conversion to ferrous (Fe^2+^) at an acidic pH in the presence of tripyridyl-S-triazine, the Fe-TPTZ complex is formed in blue. The resulting color intensity was measured by spectrophotometry at 593 nm [30, 31].

#### 2.8.6. Determination of Hydroxyproline in Lung Tissue

The Wesner method was used to measure hydroxyproline (HYP) levels in lung tissues [32].

### 2.9. Lung Histology

Lung tissue samples were kept in 10% formalin buffer. These samples were dehydrated with alcohol and immersed in paraffin. Tissues were placed in paraffin, and 5 *μ*m sections were prepared. After staining with hematoxylin-eosin, lung tissue was examined by light microscopy.

### 2.10. Statistical Analysis

Data were analyzed by SPSS software for normality and expressed as Mean ± SEM. Statistical calculations were performed for significant differences between the experimental groups using analysis of variance (ANOVA) and Tukey post hoc test. *P* < 0.05 was considered significant.

## 3. Results

**Figure 1:**
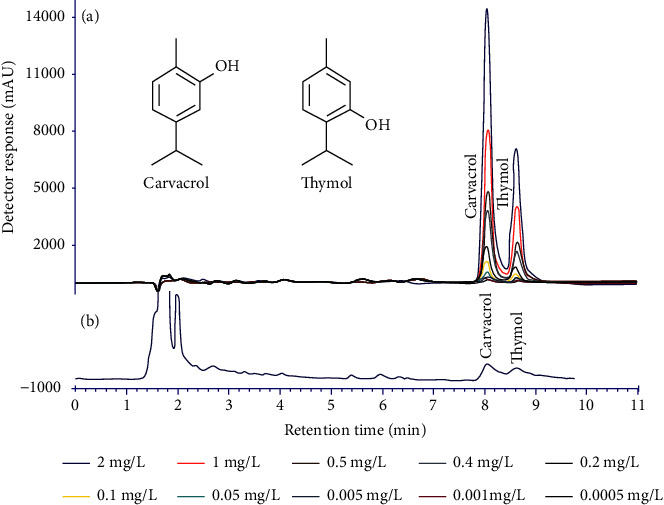
(a) Calibration curve of thymol and carvacrol. (b) HPLC chromatogram of SPHE.

Analyzed by the HPLC technique, the defatted fraction of *Stachys pilifera* aerial parts showed the presence of low-weight phenolics, thymol (55.47 ng/mL), and carvacrol (109.91 ng/mL) (Figures 1(a) and 1(b)), which is in agreement with the Asfaram et al. investigation [33].

### 3.1. Lung Tissue Oxidative Stress Markers

**Figure 2:**
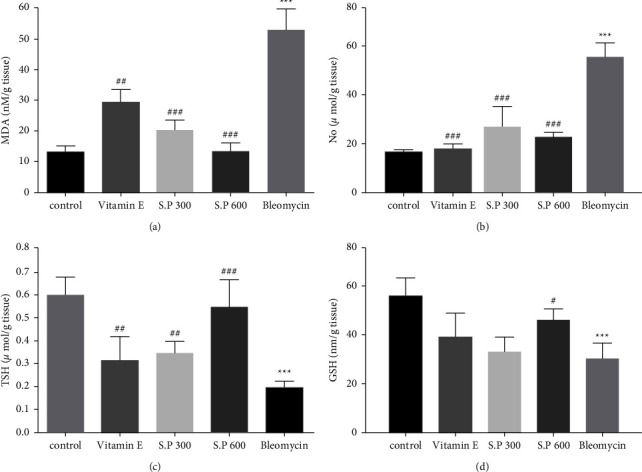
Effect of SPHE on MDA (a) NO (b) TSH (c) and GSH (d) levels in the lung tissue of BLM-induced pulmonary fibrosis rats. The results are reported as Mean ± SD, (*n* = 7). (^*∗∗∗*^*p* < 0.001, control vs Bleomycin group). (^###^*p* < 0.001, Bleomycin vs Vitamin E ^###^*p* < 0.001, Bleomycin vs S.P 300 and S.P 600) (^##^*p* <  0.01, Bleomycin vs Vitamin E ^##^*p* <  0.01, Bleomycin vs S.P 300 ^#^*p* < 0.01, Bleomycin vs S.P 600).

Our findings showed that the levels of MDA and NO in the BLM group increased significantly compared to the control group (*p*). However, using SPHE at doses of 300 and 600 mg/kg caused a substantial decrease in these indices. BLM administration significantly reduced TSH and GSH levels compared to the control group. However, in the groups receiving SPHE + BLM, these markers increased significantly compared to the BLM-treated group. Vitamin *E* treatment also had a similar effect to SPHE (Figure 2).

### 3.2. Serum Oxidative Stress Markers

**Figure 3:**
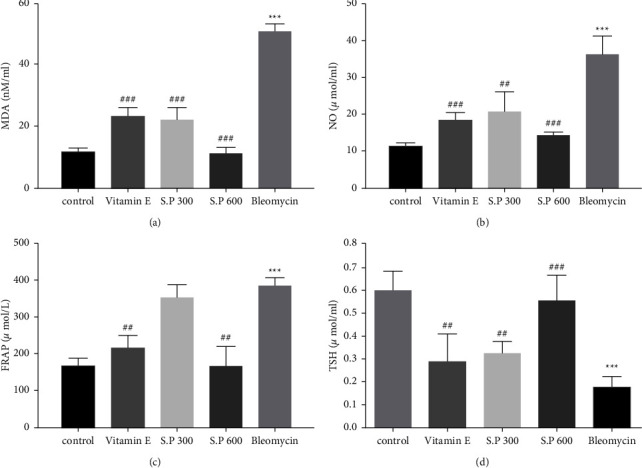
Effect of SPHE on MDA (a) NO (b) FRAP (c) and TSH (d) levels in serum of BLM-induced pulmonary fibrosis rats. The results are reported as mean ± SD, (*n* = 7). (^*∗∗∗*^*p* < 0.001, control vs Bleomycin group). (^###^*p* < 0.001, Bleomycin vs Vitamin E ^###^*p* < 0.001, Bleomycin vs S.P 300 and S.P 600) (^##^*p* <  0.01, Bleomycin vs Vitamin E ^##^*p* <  0.01, Bleomycin vs S.P 300 and S.P).

As shown in Figure 3, the MDA, FRAP, and NO levels in BLM rats were significantly increased compared to the control group. These markers were significantly reduced in the groups receiving SPHE + BLM and Vit *E*. Meanwhile, TSH levels in BLM + SPHE groups showed a significant increase compared to the BLM group.

### 3.3. Hydroxyproline Assay

**Figure 4:**
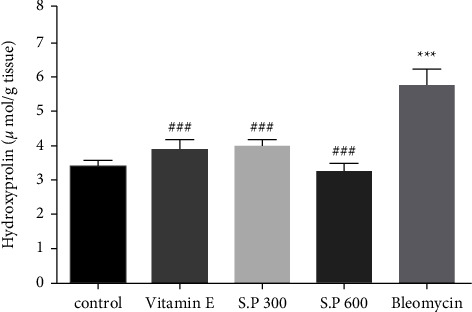
Effect of SPHE on hydroxyproline content in the liver tissue of BLM-induced pulmonary fibrosis rats. The results are reported as mean ± SD, (*n* = 7). (^*∗∗∗*^*p* < 0.001, control vs Bleomycin group). (^###^*p* < 0.001, Bleomycin vs Vitamin E ^###^*p* < 0.001, Bleomycin vs S.P 300 and S.P 600).

It was observed that BLM increased the level of hydroxyproline, which was significant compared to the control group (*p*). However, SPHE significantly reduced hydroxyproline when compared to the BLM group alone (Figure 4).

### 3.4. Histological Changes

**Figure 5:**
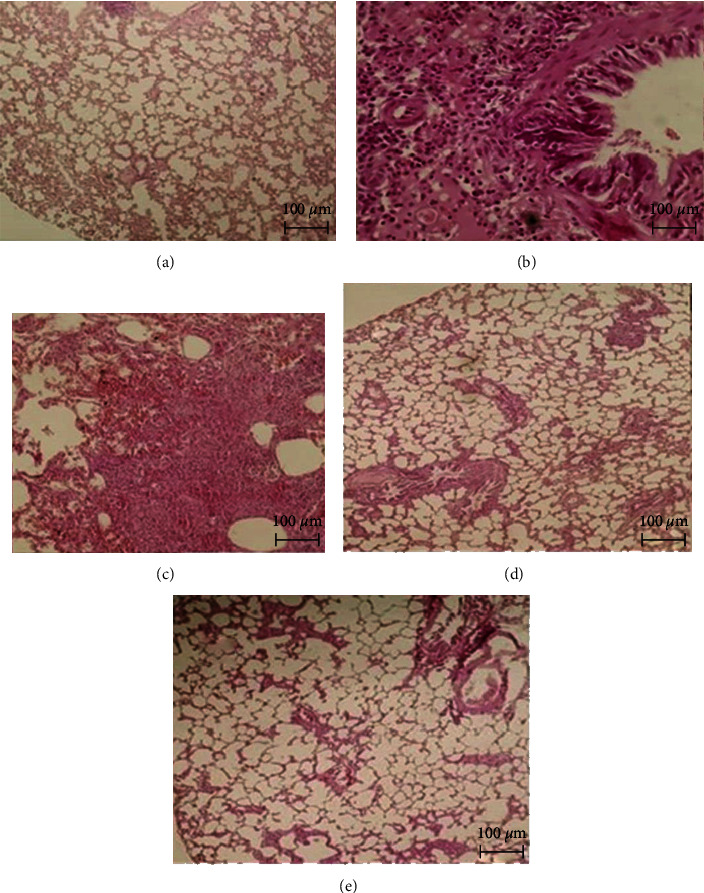
Photomicrograph of rat lung stained with hematoxylin and eosin (×10). (a) Control rat. (b) BLM rat. (c) Vit E rat. (d) BLM rat treated with SPHE (300 mg/kg). (e) BLM rat treated with SPHE (600 mg/kg).

In the control group, the cellular structures of pulmonary bronchioles and alveoli were normal. In the bleomycin group, tissue necrosis, severe hyperplasia, and alveolar deterioration were observed compared with the control group. In the SPHE + BLM group, ductal hyperplasia and moderate alveolar destruction occurred, but no tissue necrosis, inflammation, or pulmonary infiltration was observed (Figure 5).

## 4. Discussion

BLM is employed as a chemotherapeutic drug for treating various cancers. It can damage DNA on both the single-strand and double-strand levels, prevent DNA synthesis, stop cancer cells from proliferating, and trigger apoptosis pulmonary fibrosis (PF) is one of the most important of its side effects. This compound damages lung tissue by increasing extracellular matrix remodeling and redox imbalance [34]. These destructive changes provide a model for assessing pulmonary fibrosis in laboratory animals. Thus, it is possible to comprehend their potential therapeutic benefits and novel therapeutic approaches by investigating how various substances, including novel drugs and herbal medicines, affect this model. Reference [35].

Medicinal plants and their bioactive phenolic and flavonoid compounds have gained more attention due to their beneficial properties, such as antioxidant and anti-inflammatory potencies [36].

The present study investigated the possible protective effects of SPHE against the harmful effects of BLM on pulmonary tissue. In a manner similar to IPF, BLM destroys alveolar epithelial cells and provokes a pronounced inflammatory response. Reference [37]. It is assumed that lung damage can be seen in this model on 7 to 14 days, which begins with oxidative and inflammatory events and ends with fibrotic damage [6].

Based on our results, it was determined that BLM caused significant hyperplasia and alveolar damage in the animals. BLM administration dramatically elevated the concentrations of hydroxyproline (HYP), MDA, and NO, and reduced TSH and GSH levels. Oral treatment with SPHE alleviated BLM-induced pulmonary damage by restoring lung tissue architecture and normalizing the indicated oxidative stress markers.

The inflammatory mediators' cascade was activated following inflammatory cell infiltration in the early stages of pulmonary fibrosis. In the later stages, fibroblasts activate and stimulate collagen secretion [34]. As a result of excessive collagen deposition and remodeling processes, the lung tissue architecture is destroyed, and fibrosis results. It has been reported that approximately 13.14% of collagen fibers are hydroxyproline, so its amount in the tissue is a hallmark of the collagen level and the PF severity [32].

In accordance with Bahri et al. [35] and Huang et al. [34] studies, intratracheal administration of BLM markedly increased the levels of *f* HYP in lung tissue compared to the saline-treated group. Treatment with SPHE significantly reduced the elevation of HYP levels in the lung tissue due to BLM intoxication.

This activity could be attributed to phenolic and flavonoid ingredients in SPHE that produce anti-inflammatory and antioxidant effects [15]. In this line, our previous studies have reported anti-inflammatory activities and antioxidant properties of SPHE [38]. Histological examination of the lung tissues exhibited that SPHE could suppress the accumulation of collagen, duct hyperplasia, alveolar degeneration, and penetration of inflammatory cells due to BLM lung toxicity. One of the characteristics of cancer treatment and pulmonary fibrosis is weight loss. Reference [37]. Intratracheal delivery of BLM during the study decreased animal weights and increased body lung index, which is consistent with earlier findings. Reference [25]. These changes may be induced by side effects of BLM on appetite and protein catabolism, consistent with the previous studies [20].

The critical role of oxidative stress during the PF process has been demonstrated. Increased ROS and oxidant/antioxidant imbalances have been shown to play a critical role in lung tissue damage in IPF patients [39, 40]. The activation of transcription factors, cytokines, and apoptosis are some ways ROS contribute to worsening IPF [1]. In addition, ROS interferes with the extracellular matrix degradation process [34]. Thus, targeting oxidative stress processes can be considered a therapeutic approach to managing PF [4]. BLM stimulates the overproduction of ROS in animal models of PF [1]. In the presence of a compromised antioxidant system, free radicals attack saturated fatty acids in the cell membrane, which leads to lipid peroxidation and the formation of MDA [41]. In this context, it has been reported that various respiratory conditions increase MDA levels in the body fluids of patients. Reference [42]. According to our results, MDA serum and pulmonary levels increased in the group treated with BLM. It was consistent with previous studies, which showed that MDA levels in BLM-treated animals were significantly higher than in saline-treated animals. Reference [40], A significant reduction in MDA levels was observed after SPHE treatment.

There is increasing evidence that NO plays an essential role in the pathogenesis of numerous lung diseases. An isoform of NO called iNOS is responsible for producing cytokines in lung diseases. This isoform is involved in the pathogenesis of PF by producing ROS such as peroxynitrite and nitrogen dioxide. A significant increase in NO levels was observed in the BLM-treated group compared with the control group, which was consistent with the results reported by Kseibati et al. [11]. In the SPHE receiving groups, NO was significantly reduced. There was good agreement between this finding about SPHE and previous studies of the plant [14, 16, 20].

SPHE-treated groups had lower FRAP levels than BLM-treated groups, which is in line with our previous findings. As antioxidants, the thiol groups of proteins and glutathione play a significant role in inhibiting ROS and RNS. Oxidative stress reduces -SH reserves by oxidizing the -SH groups into disulfide or sulfonic acid. References [43, 44]. Furthermore, the relationship between a decrease in GSH and an increase in ROS in the BLM-induced pulmonary fibrosis animal model has been confirmed [1]. In agreement with the study of Gamed et al., the levels of GSH and TSH in the BLM group were significantly lower than in the control group. Reference [40]. The Results showed that GSH and TSH levels rose significantly in SPHE-treated groups.

However, Vit *E* as an antioxidant behaved similarly to SPEH in the evaluated parameters, but it did not significantly affect GSH levels. Polyphenols such as resveratrol, mangiferin, quercetin, and dihydroquercetin mitigate lung damage after BLM induction in animal models [31]. It is also reported that quercetin and gallic acid restore lung SOD and GSH levels while decreasing the inflammation factors, NO, and IL-6 levels in BLM-induced pulmonary fibrosis in rats [32]. Carvacrol and thymol are two of the essential components of SPHE. Several studies have demonstrated the antioxidant effects, free radical scavenging properties, anti-inflammatory properties, and beneficial properties of the compounds in various animal disease models [45–47]. Salimoglu Sen et al. found that preadministration of carvacrol or pomegranate extract reduced MDA levels, total oxidant status, and oxidative stress index in methotrexate-induced lung injury rats. Reference [24]. Hence, polyphenol ingredients of SPHE such as carvacrol and thymol may be partially responsible for the protective effect on BLM-induced fibrosis.

Future research could address two significant limitations of the study. First, SPHE is also examined for its effects on apoptosis and inflammation in BLM-induced lung fibrosis. The second step is to prepare and evaluate the extract's different fractions or principal components.

## 5. Conclusion

The evidence from this study suggests SPHE on BLM-induced fibrosis. Histological examination of lung tissue confirmed the biochemical results. Thus, it can be postulated that a defatted SPHE involving thymol and carvacrol as the significant constituents may effectively reduce BLM damage due to its antioxidant effects. More research is required to determine the precise mechanisms of SPHE in managing pulmonary fibrosis.

## Data Availability

The data used to support the findings of this study are included within the article. The raw materials of data are available from the corresponding author upon reasonable request.
